# Successful therapy with tenofovir disoproxil fumarate (TDF) in patients with chronic hepatitis B (CHB) does not guarantee amelioration of liver damage assessing by transient elastography. A retrospective - prospective multicenter study

**DOI:** 10.1186/s12876-024-03200-3

**Published:** 2024-04-12

**Authors:** Hariklia Kranidioti, Konstantinos Zisimopoulos, Theodora Oikonomou, Theodoros Voulgaris, Spyros Siakavellas, Polixeni Agorastou, Melanie Deutsch, Christos Triantos, Ioannis Goulis, George Papatheodoridis, Spilios Manolakopoulos

**Affiliations:** 1 2nd Academic Department of Internal Medicine, Liver– GI Unit, General Hospital of Athens “Hippocration”, National and Kapodistrian University of Athens, 114 Vas. Sofias str, 11527 Athens, Greece; 2 Department of Gastroenterology, University Hospital of Patras, Patra, Greece; 3 4thDepartment of Internal Medicine, General Hospital of Thessaloniki “Hippocration”, Aristotelion University of Thessaloniki, Thessaloniki, Greece; 4 Academic Department of Gastroenterology, General Hospital of Athens “Laiko”, National and Kapodistrian University of Athens, Athens, Greece

**Keywords:** Liver stiffness, Chronic hepatitis B, TDF, BMI, Metabolic factors

## Abstract

**Background:**

Preventing disease progression and viral suppression are the main goals of antiviral therapy in chronic hepatitis B (CHB). Liver stiffness measurement (LSM) by transient elastography is a reliable non-invasive method to assess liver fibrosis in patients with CHB. Our aim was to explore factors that may affect changes in LSMs during long term tenofovir (TDF) monotherapy in a well characterized cohort of patients with compensated CHB.

**Methods:**

We analyzed serial LSMs in 103 adult patients with CHB who were on TDF monotherapy and had at least three LSMs over a period of 90 months.

**Results:**

Twenty-five (24%) patients had advanced fibrosis at baseline. A significant decline in mean LSM between baseline and last visit (8.7 ± 6.2 kPa vs. 6.7 ± 3.3, *p* = 10^− 3^) was observed. Twenty-four (23%) patients had progression of liver fibrosis with mean increase in liver stiffness of 2.8 kPa (range: 0.2–10.2 kPa). Multivariate analysis showed that BMI ≥ 25 (OR, 0.014; 95% CI, 0.001–0.157; *p* = 0.001) and advanced fibrosis (OR, 5.169; 95% CI, 1.240–21.540; *p* = 0.024) were independently associated with a fibrosis regression of > 30% of liver stiffness compared to baseline value.

**Conclusions:**

In CHB patients TDF monotherapy resulted in liver fibrosis regression, especially in patients with advanced fibrosis. Despite the successful antiviral effect of TDF, 1 out of 4 patients had liver fibrosis progression. Obesity and advanced fibrosis at baseline were independently associated with significant liver fibrosis regression.

## Background

Chronic hepatitis B (CHB) remains a serious global problem and leading cause of cirrhosis and hepatocellular carcinoma (HCC) accounting for more than 800,000 deaths per year [1]. Long term monotherapy with entecavir, tenofovir disoproxil fumarate (TDF) or tenofovir alefanamide (TAF) has been proposed as first line treatment for patients with CHB as there is evidence supporting that maintenance of virological suppression with nucleos(t)ide analogue (NA) therapy leads in biochemical remission, histological improvement and reduction of liver related complications [2–8].

However, some patients with CHB particularly those with more advanced disease do not improve or decompensate despite complete viral suppression and may have continuing liver inflammation and scarring with progression of architectural distortions and worsening of portal hypertension [9]. Therefore, assessing evolution of liver damage and identifying patients with CHB under anti-viral therapy who may have progressive disease remains clinically important.

Liver stiffness measurement (LSM) by transient elastography has replaced liver biopsy in assessing liver fibrosis [10]. The method has been validated in patients with CHB and is clinical useful in evaluating disease stage, severity of portal hypertension and treatment consideration [11, 12]. Repeat LSMs during anti-viral therapy with NAs may provide useful clinical information regarding liver fibrosis progression or regression. Several studies using non– invasive methodologies including transient elastography have demonstrated fibrosis regression with long term NA therapy [13–15]. The majority however of studies were retrospective, included a rather small number of patients and had only one fibrosis assessment while recently discrepancies have been reported between liver biopsy findings and transient elastography values [16].

The aim of this study was to address the changes in liver stiffness (LS) assessed by transient elastography in patients with CHB receiving long-term TDF in a real-world setting and to investigate non-virological parameters that are associated with fibrosis regression or progression.

## Methods

### Patients

The REST-B study was a multicenter study included 276 patients with CHB who were on or initiated TDF monotherapy between 1st January till 31st December 2016. All participating centers were throughout Greece (1. Liver-Gastroenterology unit– Academic Department of Internal Medicine in Athens, 2. Academic Department of Gastroenterology in Athens, 3. Academic Department of Internal Medicine in Thessaloniki, 4. Gastroenterology unit - Academic Department of Internal Medicine in Patra).

REST-B study had a retrospective and prospective design:

- Patients who were already on monotherapy with TDF during the year 2015, were the retrospective group who started TDF before 31st Dec 2015.
- Patients who initiated TDF monotherapy between 1st January till 31st December 2016, were the prospective group.

Current study population was a sub-cohort of the REST-B population. (The results of REST-B data have been published [17, 18].

Inclusion criteria were:

- age > 18 years old.
- CHB HBeAg positive or HBeAg negative.
- Compensated liver disease.
- Continuous TDF monotherapy for at least 3 years.
- At least 3 reliable liver stiffness measurements (LSMs) (baseline and ≥ 2 measurements during follow-up, at least 12 months apart). Baseline was considered day of TDF initiation.

We excluded patients with HCV, HDV or human immunodeficiency virus co-infections, pregnancy at baseline or during follow up and those who consumed > 30 gr alcohol daily. Patients with HCC or decompensation occurrence during follow up were included only when two LSMs were performed before development of the complication. We also excluded patients with a history of liver transplantation and significant medical comorbidities including congestive heart failure which make accurate transient elastography difficult.

The study was approved by the Ethics committee of the participating centers and was conducted according to the principles of the Declaration of Helsinki. The requirement for informed consent was waived by the Ethics Committee of the centers because we collected ordinary clinical data in the majority of the patients retrospectively.

All patients were followed by their physicians at the outpatient clinics every 6 months with clinical (physical examination, vital signs) and laboratory (complete blood count, creatinine, transaminases, phosphorus levels) assessments according to local or international clinical practice guidelines. Serum HBV DNA levels were determined every 6 to 12 months. The medical, smoking, medication and anthropometric measurements were assessed and recorded at the initiation of antiviral treatment (considered as baseline) and according to clinician judgement during follow up. NA dosing was adjusted according to renal function tests when appropriate. All patients with cirrhosis or with PAGE-B score above 10 were advised to have ultrasonography with or without alpha fetoprotein measurements every 6 months. We followed patients to death, HCC, decompensation or switch/stop TDF therapy.

### Transient elastography (ΤΕ)

We performed ΤΕ for liver stiffness estimation (Fibroscan, Echosens, Paris, France). Τhe technique of TE has been previously described, while correlation with liver fibrosis has been studied and validated in CHB. All LSMs were performed by experienced operators with a prior experience of more than 500 measurements. Liver stiffness measurements (LSMs) were expressed as the median value of at least 10 successful acquisitions in units of kilopascals (kPa). Liver stiffness measurements (LSMs) were only considered reliable with a success rate of 60% or above, combined with an interquartile range of 30% or less. The M probe was used for all measurements.

### Definitions

Advanced liver fibrosis was defined as liver stiffness (LS) ≥ 9 kPa and cirrhosis as liver stiffness > 12 kPa with ALT levels < 5 x ULN (upper limit normal = 40 IU/L). Virological remission was defined by undetectable serum HBV DNA (< 45 IU/mL) with sensitive PCR assays. Biochemical remission was defined as AST or ALT levels < 40 IU/L. Significant regression in liver stiffness was defined as reduction > 30% in LSM between baseline and last measurement. Diabetes, dyslipidemia and hypertension were defined with the current use of antidiabetic, anti-lipidemic and anti-hypertensive medication before TDF initiation respectively. Patients with one or more of the following comorbidities, diabetes mellitus, hypertension, dyslipidemia and BMI ≥ 30 kg/m^2^ were considered as individuals with metabolic syndrome. Patients with BMI ≥ 25 kg/m^2^ and ≥ 30 kg/m^2^ were defined as overweight and obese respectively. Liver decompensation included ascites, encephalopathy and bleeding from gastro-esophageal varices.

### Statistical analysis

All data were analyzed using the statistical package SPSS 25 (SPSS Inc, an IBM Company). Continuous variables are presented by their mean values ± standard deviation or median values ± interquartile range (IQR). The comparison of continuous variables between 2 independent groups of patients was performed by t-test or Mann-Whitney test and comparison between dependent groups of patients was performed by Wilcoxon Rank test. Categorical variables were summarized as frequencies and percentages. Comparisons between categorical variables were performed by corrected chi-squared or two-sided Fisher’s exact test. To identify independent predictors of fibrosis improvement, we performed univariate and subsequent multivariate regression analysis. Odds ratios (ORs) and corresponding 95% confidence intervals (CIs) are indicated. *P*-values less than 0.05 were considered as statistically significant.

## Results

### Demographic characteristics

103 CHB patients from the REST-B cohort were included in the analysis; 36 started TDF during 2016 while 67 had initiated before 31st December 2015. The mean age was 56.7 ± 13 years old, 58 (56%) were males and 8 (7.8%) were HBeAg positive; median follow-up period was 90 (range: 36–154, SD: 30) months, 93 (90%) patients had ≥ 48 months and 80 (78%) ≥ 60 months of follow-up. At the time of TDF initiation 44 (43%) patients were above 60 years old, 25 (24%) patients had advanced fibrosis / cirrhosis [11 of them had advanced fibrosis (stiffness 9-12 kPa) and 14 cirrhosis (> 12 kPa)], 21 (20%) were active smokers, 29 patients (28%) had hypertension, 10 (10%) diabetes mellitus, 11 (11%) had dyslipidemia using statin therapies, 46 (45%) had BMI ≥ 25 kg/m^2^ and 10 (10%) had BMI ≥ 30 kg/m^2^. Two patients with ALT > 5x ULN and very high LSM at baseline (22 and 30 kPa) were considered cirrhotic although they did not fulfill the definition. At baseline, 46% of the patients had at least one and 10% had two of the following metabolic risk factors: hypertension, diabetes mellitus, dyslipidemia or BMI ≥ 30 kg/m^2^ (Table 1).

**Table 1:** Baseline (at the initiation of TDF treatment) characteristics of the 103 patients

Baseline patients’ characteristics (*N* = 103)	
Age, years* > 60 years old, n (%)	56.7 ± 13 44 (43%)
Male patients, n (%)	58 (56%)
HBeAg– positive, n (%)	8 (7.8%)
Baseline liver stiffness (kPa)*	8.7 ± 6.2
**Advanced fibrosis / cirrhosis n (%)** Cirrhosis n (%)	25 (24%) 14 (14%)
Treatment duration with TDF**, months	90 (36–154)
ALT (IU/L)**	36.9 (11-2130)
HBV DNA (log_10_ IU/L)**	4.8 (2.3–8.8)
Platelet x 10^9^ /L**	200 (57–337)
**BMI (Kg/m** ^**2**^ )* ≥ 25Kg/m^2^, n (%) ≥ 30 Kg/m^2^, n (%)	25 ± 4 46 (45%) 10 (10%)
Diabetes Mellitus, n (%)	10 (10%)
Hypertension, n (%)	29 (28%)
Dyslipidemia, n (%)	11 (11%)
Metabolic syndrome, n (%)	47 (46%)
Active smokers, n (%)	21 (20%)

*mean ± SD, ** median (range)

### Clinical remission

Virological remission rates were 96% and 98.6%, while ALT was within normal range (< 40 IU/L) in 87% and 90% of the patients at 24 and 48 months, respectively. None of the patients presented virological rebound or evidence of antiviral resistance. HBeAg seroclearance occurred in 1/8 positive patients (at 22nd month of follow up), HBsAg seroclearance in 4 patients [median 53.5 (36–93) months of follow up]. Age, gender, diabetes mellitus, BMI, hypertension, and dyslipidemia were not associated with virological or biochemical response to therapy. There were no deaths during follow up. None of the patients developed decompensation, while hepatocellular carcinoma was diagnosed in two patients (64 and 72 months after initiation of TDF).

### Liver stiffness

**Fig. 1:**
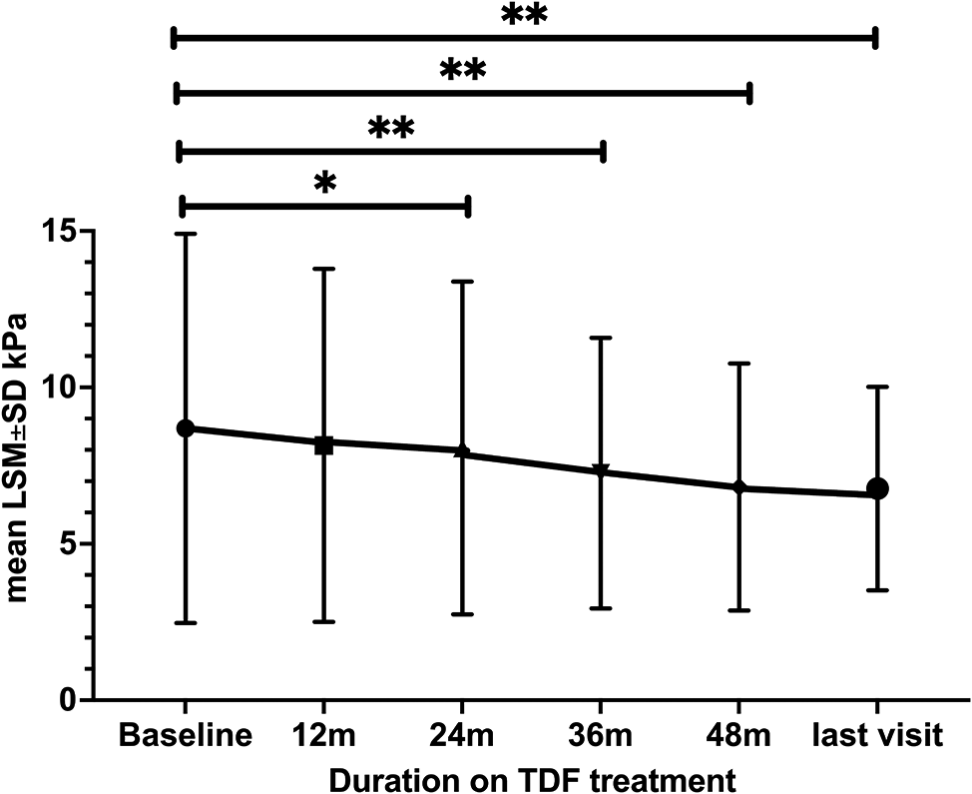
Mean LSM levels of the 103 patients at baseline, 12, 24, 36 months and of 93 patients who have reached 48 months and 80 patients at last visit (≥ 60 months)

Mean baseline LS was 8.7 ± 6.2 kPa. Eighty-two (79.6%) patients had LSM < 10 kPa, 14 (13.6%) 10-20 kPa and 7 (6.8%) > 20 kPa. Twenty-five (24%) patients had advanced fibrosis/cirrhosis with a mean LSM 16.5 ± 8 kPa vs. 6.1 ± 1.4 kPa for those without advanced fibrosis/cirrhosis. A significant progressive reduction in mean LSM between baseline and 24, 36, 48 months and last visit was observed (8.7 ± 6.2 kPa vs. 8 ± 5 *p* = 0.02, 7.2 ± 4.3 *p* < 10^− 3^, 6.8 ± 4 *p* = 0.005, 6.7 ± 3.3 *p* = 10^− 3^, respectively) (Fig. 1).

**Fig. 2:**
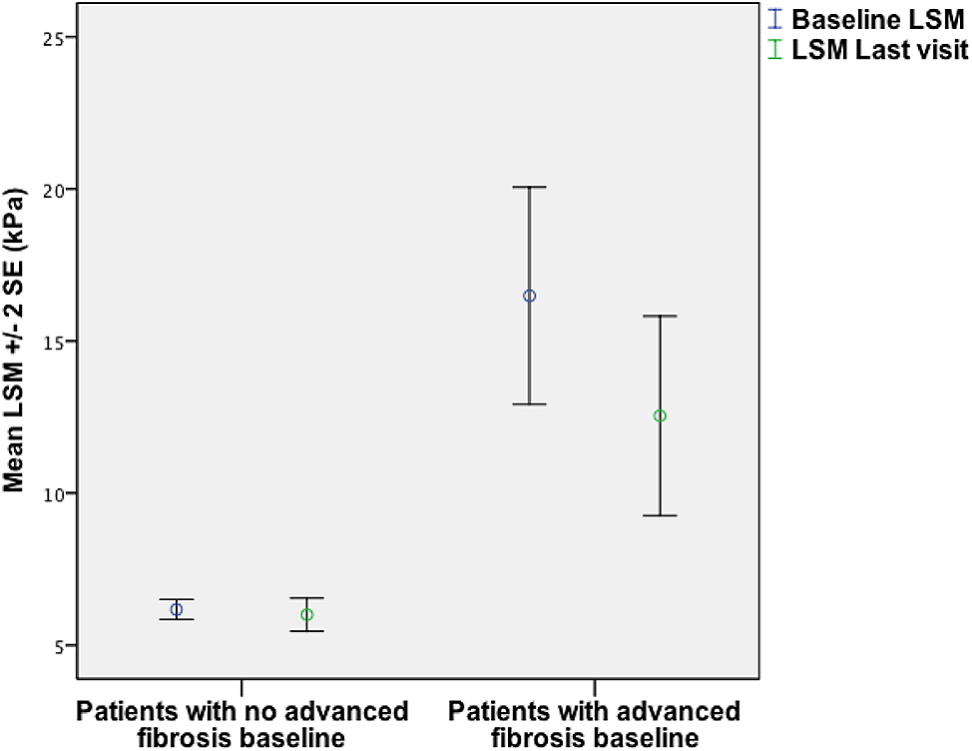
LSM baseline and at the last visit of the patients with advanced fibrosis (≥ 9 kPa) (*p* = 0.001) compared with the patients with no advanced fibrosis (*p* = 0.01)

At the last visit, 72.5% of the patients showed a reduction in LSM with 33% and 38% of the patients achieving more than 30% and 20% reduction respectively. The mean reduction of LSM was 2.2 kPa (range: 2-15.5 kPa) and was higher in patients with than without advanced fibrosis/cirrhosis at baseline (3.9 vs. 0.5 kPa, *p* < 0.0001) (Fig. 2). The proportion of patients with advanced fibrosis/cirrhosis decreased from 24% at baseline to 19% at last visit. Of the 25 patients with advanced liver fibrosis/cirrhosis at baseline, 50%, 23% and 27% had LS < 10 kPa, 10-20 kPa and > 20 kPa respectively at last visit.

Characteristics of the patients who presented or not significant regression in liver stiffness (> 30% reduction in LSM) are presented in Table 2. Baseline LS, the percentage of the patients with advanced fibrosis/cirrhosis, BMI and the percentage of the overweighted patients were represented with significant difference between the two groups (Table 2).

**Table 2:** Baseline (at the initiation of TDF treatment) characteristics of the 103 patients stratified by significant liver stiffness regression achievement after 90 (range: 36–154) months TDF monotherapy

Baseline patients’ characteristics	Stiffness regression (*N* = 34)	No stiffness regression (*N* = 69)	p
Age, years* > 60 years old, n (%)	57 ± 13 16 (47%)	55 ± 14 28 (40%	0.5 0.6
Male patients, n (%)	18 (53%)	40 (58%)	0.6
HBeAg– positive, n (%)	3 (10%)	5 (7%)	0.7
Baseline liver stiffness (kPa)*	10 ± 7	8 ± 5	0.01
**Advanced fibrosis/cirrhosis** Cirrhosis n (%)	14 (40%) 8 (23%)	11 (16%) 6 (9%)	0.02 0.09
Treatment duration with TDF*, months	79 ± 26	79 ± 27	0.9
ALT (IU/L)** ALT > 40 IU/mL, n (%)	43 (14–458) 17 (50%)	30 (11-2130) 31 (45%)	0.3 0.7
HBV DNA (log_10_ IU/L)**	4.9 (2.7–7.4)	4.7 (2.3–8.8)	0.7
Platelet x 10^9^ /L**	196 (61–337)	204 (68–334)	0.06
**BMI (Kg/m** ^**2**^ )* ≥ 25Kg/m^2^, n (%) ≥ 30 Kg/m^2^, n (%)	24 ± 5 1 (3%) 0	27 ± 3 45 (65%) 10 (14%)	0.001 < 0.001 0.09
Diabetes Mellitus, n (%)	3 (8%)	7 (10%)	1
Hypertension, n (%)	14(41%)	15(21%)	0.1
Dyslipidemia, n (%)	1 (3.7%)	10 (15%)	0.3
Parameters of metabolic syndrome, n (%)	17 (50%)	30 (43%)	0.6
Active smokers, n (%)	9 (25%)	12 (18%)	0.6

*mean ± SD, ** median (range)

Twenty-four (23%) patients presented LS increase between baseline and last visit; the mean increase was 2.8 kPa (range: 0.2–10.2 kPa). We did not find any significant difference in age, BMI and the presence of metabolic risk factors between the regressor and progressor groups.

### Liver stiffness and metabolic risk factors

Patients with CHB who presented metabolic risk factors compared to those without had significant higher mean LSM at baseline (10.5 ± 8.2 vs. 7.6 ± 4.4 kPa, *p* = 0.02). 65% of the patients with metabolic factors at baseline had LSM < 10 kPa compared with 89% of the patients without (*p* = 0.02).

A non-significant reduction in LSM values was observed in patients with metabolic risk factors (baseline: 10.5 kPa vs. last visit: 9.1 kPa, *p* = 0.1); in contrary, we observed significant reduction in LSM values in patients without any risk factor (baseline: 7.6 kPa vs. last visit: 6.6 kPa, *p* = 0.001). Patients with BMI ≥ or < 25 did not present any difference in LSM at baseline (8.6 kPa vs. 8.3 kPa, *p* = 0.7) but they had significant difference at the last visit (8.6 kPa vs. 7.1 kPa, *p* = 0.01).

**Fig. 3:**
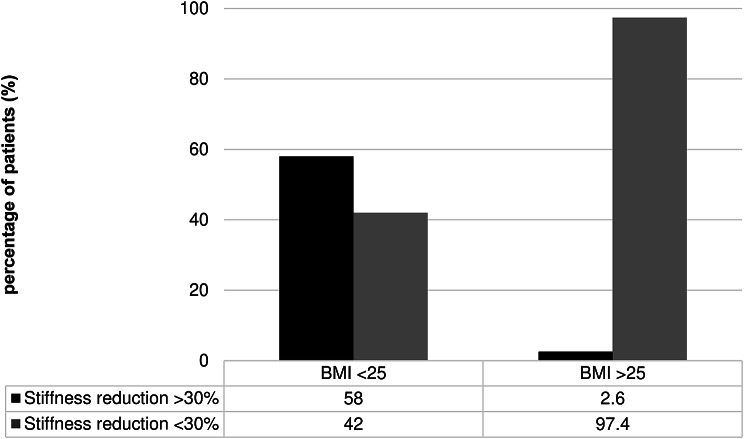
Rate of the patients with BMI ≥ or < 25 who presented or not significant fibrosis regression

**Fig. 4:**
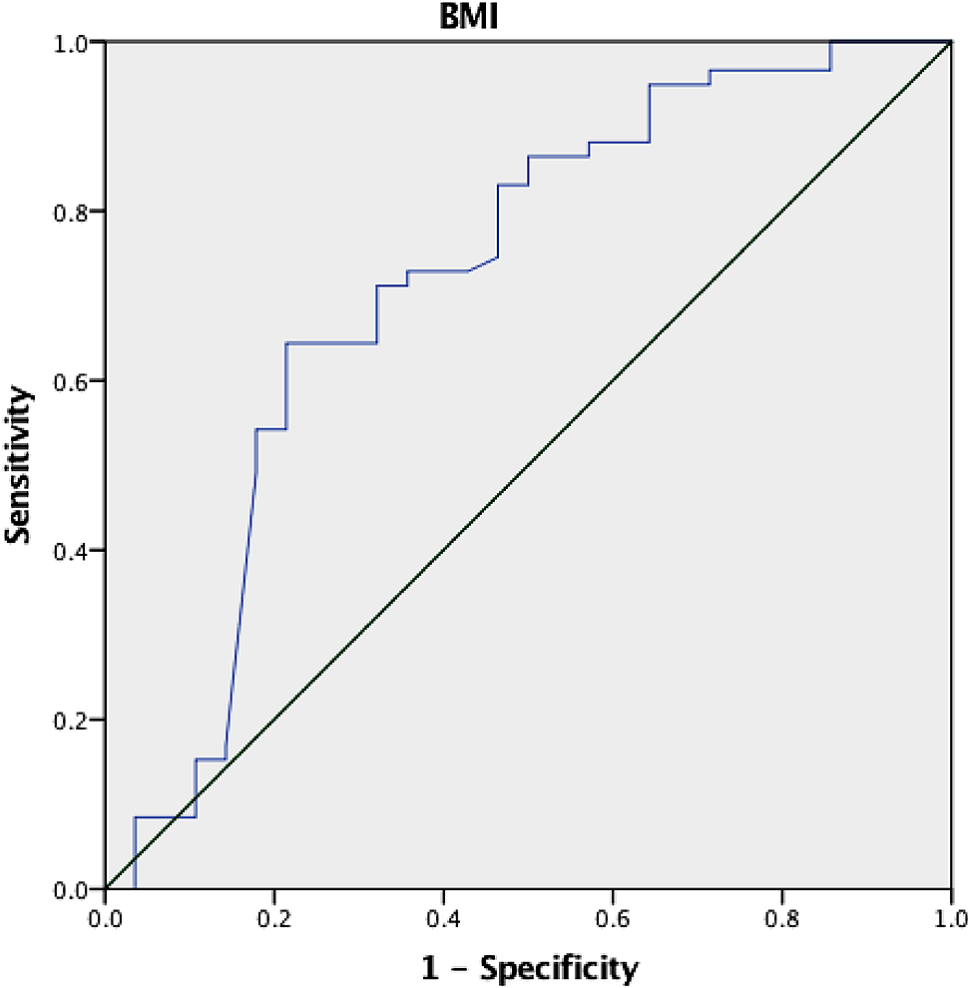
Receiver operating curve of the body mass index for prediction of liver significant fibrosis regression. AUROC of 0.72 (95% CI, 0.593–0.844; *p* = 0.001)

Significant liver stiffness regression was observed in 58% of the patients with BMI < 25 vs. 2.6% of those with BMI ≥ 25 (*p* < 0.0001) (Fig. 3). None of the patients with BMI ≥ 30 achieved significant regression in liver stiffness. The optimal BMI for prediction of no significant liver stiffness regression was estimated as 27 with an AUROC of 0.72 (95% CI, 0.593–0.844; *p* = 0.001) (Fig. 4).

Univariate logistic regression analysis indicated that factors associated negatively with significant liver stiffness regression by LSM were BMI (*p* = 0.006) and BMI ≥ 25 (*p* = 0.0001) while advanced fibrosis/cirrhosis at baseline was associated positively (*p* = 0.013). Multivariate logistic regression analysis revealed that BMI ≥ 25 (OR, 0.014; 95% CI, 0.001–0.157; *p* = 0.001) and advanced fibrosis/cirrhosis (OR, 5.169; 95% CI 1.240–21.540; *p* = 0.024) were associated with significant liver stiffness regression (Table 3).

**Table 3:** Baseline (at the initiation of TDF treatment) characteristics associated with significant liver stiffness regression achievement after 90 (range: 36–154) months TDF monotherapy

	Univariate		Multivariate	
Variables	P value	**OR (95% CI)**	P value	**OR (95% CI)**
Baseline LSM	0.106	1.066 (0.986–1.153)		
Advanced fibrosis/cirrhosis Cirrhosis	0.013 0.015	3.63 (1.310-10.1058) 14.824 (1.687-130.248)	0.024 0.811	5.169 (1.240–21.540) 1.356 (0.112–16.399)
Baseline ALT	0.627	1 (0.999–1.002)		
Baseline HBV DNA	0.883	1(1–1)		
Platelet x 10^9^ /L	0.031	1 (1–1)		
Age	0.419	1.013 (0.981–1.047)		
> 60 years old	0.54	1.312 (0.542–3.177)		
Males	0.65	0.816 (0.338–1.972)		
Parameters of metabolic syndrome	0.238	0.583 (0.238–1.428)		
BMI BMI ≥ 25	0.006 0.0001	0.830 (0.727–0.947) 0.019 (0.002–0.146)	0.592 0.001	0.949 (0.783–1.149) 0.014 (0.001–0.157)
Hypertension	0.5	1.382 (0.502–3.810)		
Dyslipidemia	0.16	0.216 (0.026–1.828)		
Diabetes	0.814	0.815 (0.148–4.475)		
Smoking	0.444	1.533 (0.513–4.584)		

## Discussion

We studied changes in LS using transient elastography before and at several time points of TDF monotherapy in a well characterized cohort of 103 adult Caucasian patients with compensated CHB. Our prospectively collected data further confirmed that successful long term TDF therapy had a beneficial effect in liver fibrosis as we observed LSM values to decline in 3 out 4 of patients. In addition, we found that increased body adiposity as presented by BMI hampers the significant liver stiffness regression and that BMI above 27 indicates the point where the beneficial effect of TDF therapy on liver fibrosis is lost.

Several studies and meta-analysis have presented liver fibrosis regression with oral antiviral therapy [15]. The results however are inconclusive in regards of the magnitude of decline while limited information exists for course of decline, the proportion of progressors and the clinical outcome of patients. The retrospective design of the studies, the limited number of LSMs and the short follow up might be the explanations for these discrepancies. In the present study we analyzed serial LSMs using the only reliable and well validated non-invasive method [19] for liver fibrosis assessment in patients with CHB, offering the evidence to answer critical and remaining gaps in existing literature. We observed that the mean liver stiffness was reduced from 8.7 kPa to 6.7 kPa after 90 months of therapy. Liver stiffness reduction was observed in 72.5% of our patients with 33% to have more than 30% reduction from baseline. The results are in line with those of the meta-analysis by Facciorusso et al. [15] who presented data from 24 studies (22 from Asia) including 2228 patients. In both studies the stiffness regression was progressive and incremental over time. However, contrary to our data Facciorusso et al. [15] reported higher stiffness reduction after 2nd year of therapy; we observed mean stiffness decline 2 kPa at 3 and 4 years compared to 4.15 kPa in the meta-analysis. Including patients with high transaminase levels, more advanced liver disease at baseline and the limited number of LSMs during follow up could be the main explanations for the discrepancy. Although difficult to make direct comparison our results are similar with those of the unique study by Marcellin et al. [6] who demonstrated significant fibrosis regression at 5th year liver biopsies in a 51% of 348 patients with CHB who were on TDF maintenance therapy. Moreover, a recent retrospective study [20], with 337 Asian CHB patients under NA therapy, addressed that at follow up liver biopsy (> 2 years NA therapy) 50% of the patients had liver fibrosis regression. Also, the same study justifies our choice for the definition of significant regression in liver stiffness as a reduction > 30% in LSM between baseline and last measurement. The authors proved that a decrease in LSM 25% is the optimal cutoff for predicting liver fibrosis regression by at least one stage assessed by METAVIR scoring system (biopsy).

Noteworthy according to our data liver stiffness regression occurred at different degrees at different group of patients. Patients with advanced fibrosis or cirrhosis have had pronounced regression while some others had minimal regression. One explanation could be that the beneficial effect of antiviral therapy could be more obvious in patients with severe fibrosis. Furthermore, patients with mild to moderate fibrosis could not have large-scale differences in the LSMs, while patients with advanced fibrosis had a greater spectrum to prove their LS improvement. Importantly, half of the patients with advanced fibrosis/cirrhosis at baseline had LS < 10 kPa at the last visit and may have negligible risk for liver related morbidity and mortality [21]. Chon et al. [22] analyzed annual LSMs in 120 patients with advanced fibrosis/cirrhosis under NAs and in contrast to our results reported that low baseline stiffness was significant predictor for 5 years fibrosis improvement. The different selection criteria and design of the study may explain this discrepancy. On the contrary to the above study and in agreement with our results, an Asian prospective study [23] demonstrated recently that a higher baseline LSM was the single independent predictor for the significant (> 30%) LSM reduction in CHB patients under NA therapy.

Another interesting finding of our study is that ¼ of our patients had progressive liver disease. As serum HBV DNA was undetectable in > 90% of our patients for long periods, our finding supports that virological suppression is not equal to fibrosis improvement and that other factors may be implicated in liver damage. Patients with metabolic factors presented higher values of liver stiffness and non-significant reduction during the follow-up. Overweight and obese patients had significantly higher mean LSM at the last visit compared to the patients with BMI < 25, while only 2.6% of patients with BMI > 25 had > 30% reduction in LSM compared to baseline values and none with BMI ≥ 30. According to our data the threshold of BMI 27 could be used as predictor of no significant liver stiffness regression despite the virological response and may indicate closer follow up. Similar results have been reported from 2 Asian studies [24, 25] which however included only patients with advanced fibrosis.

Hepatic steatosis could be a confounding factor which leads to liver stiffness overestimation according to several studies [26, 27]. However, the aim of our study was not the diagnostic accuracy of transient elastography in patients with or not steatosis. We wanted to address the progression or regression of LSM as a representative surrogate marker of liver fibrosis. Unfortunately, we do not have measurements of hepatic steatosis with CAP (Controlled Attenuation Parameter). Regarding, the stage of steatosis as it is provided by Ultra Sound was not always addressed and was not performed by the same radiologist.

The progression of fibrosis to cirrhosis and portal hypertension is the main milestone in the evolution of chronic liver disease. Therefore, our findings underline the importance of performing LSMs during therapy with NAs and in addition added information regarding factors associated with progression of fibrosis despite successful antiviral therapy in cases with CHB. The significance of the magnitude of decline and the association with the threshold of LSM that recent Baveno [21] suggest regarding cACLD will be a matter of further research.

One may argue that APRI and FIB-4 were not used in our study. Chon et al. [22] declared that these scores had high correlation with LSM values at baseline, and these markers decreased in a linear fashion, but not significantly every year, during antiviral treatment. However, a study [28] included 575 patients with CHB reported that APRI and FIB-4 scores are not suitable for assessing fibrosis especially in those receiving antiviral therapy. Furthermore, a recent retrospective cohort study, with 337 CHB patients who underwent paired liver biopsy and NITs (non-invasive tests), declared that only LSM might be used to monitor regression of liver fibrosis during antiviral treatment using NAs [20].

The limitations of our study were the small number of patients and the absence of detailed information about metabolic parameters as levels of triglycerides or HDL-cholesterol, HbA1c values and evidence of prediabetes. The strengths of our study were the prospective design, the long duration of follow-up and the use of strict criteria for the definition of significant regression in liver stiffness as the reduction > 30% in LSM which is probably able to represent a histological improvement.

## Conclusions

Our data confirmed that long-term TDF monotherapy in CHB patients is associated with significant liver stiffness improvement. The beneficial effect of TDF is more pronounced in patients with advanced fibrosis/cirrhosis. However, BMI ≥ 25 ameliorated the liver stiffness improvement. Our data supports that lifestyle modification and BMI reduction should be recommended in all CHB patients.

Metabolic dysfunction-associated steatotic liver disease (MASLD) is a new “international scourge”. In the future, it may represent a common comorbidity of the chronic hepatitis B patients, that should be taken under consideration for their effective management and follow-up. Further research is needed in order to provide insights in the interplay between the metabolic risk factors, hepatic steatosis and chronic hepatitis B.

## Data Availability

The datasets used and/or analysed during the current study are available from the corresponding author on reasonable request.

## Abbreviations

ALT: Alanine Transaminase
AST: Aspartate Transaminase
BMI: Body Mass Index
cACLD: compensated Advanced Chronic Liver Disease
CHB: Chronic Hepatitis B
HCC: Hepatocellular carcinoma
HCV: Hepatitis C virus
HDV: Hepatitis D virus
LS: Liver Stiffness
LSM: Liver Stiffness Measurement
NA: Nucleos(t)ide Analogue
NITs: Non-Invasive Tests
TAF: Tenofovir Alafenamide
TDF: Tenofovir Disoproxil Fumarate
TE: Transient Elastography
ULN: Upper Limit Normal
